# Pear pomace soluble dietary fiber ameliorates the negative effects of high-fat diet in mice by regulating the gut microbiota and associated metabolites

**DOI:** 10.3389/fnut.2022.1025511

**Published:** 2022-10-20

**Authors:** Yuehong Ji, Kemin Mao, Jie Gao, Bimal Chitrakar, Faizan Ahmed Sadiq, Zhongxuan Wang, Jiangna Wu, Chao Xu, Yaxin Sang

**Affiliations:** ^1^College of Food Science and Technology, Hebei Agricultural University, Baoding, China; ^2^Flanders Research Institute for Agriculture, Fisheries and Food (ILVO), Technology and Food Sciences Unit, Melle, Belgium

**Keywords:** soluble dietary fiber, gut microbiota, untargeted metabolomics, isobutyryl carnitine, *Akkermansia*, obesity

## Abstract

The gut microbiota and related metabolites are positively regulated by soluble dietary fiber (SDF). In this study, we explored the effects of SDF from pear pomace (PP) on the regulation of gut microbiota and metabolism in high-fat-diet-fed (HFD-fed) C57BL/6J male mice. The results showed that PP-SDF was able to maintain the HFD disrupted gut microbiota diversity with a significant increase in *Lachnospiraceae*_UCG-006, *Akkermansia*, and *Bifidobacterium* spp. The negative effects of high-fat diet were ameliorated by PP-SDF by regulating lipid metabolisms with a significant increase in metabolites like isobutyryl carnitine and dioscoretine. Correlation analysis revealed that gut microbiota, such as *Akkermansia* and *Lachnospiraceae*_UCG-006 in the PP-SDF intervention groups had strong positive correlations with isobutyryl carnitine and dioscoretin. These findings demonstrated that PP-SDF interfered with the host's gut microbiota and related metabolites to reduce the negative effects caused by a high-fat diet.

## Introduction

Pear (*Pyrus bretschneideri*) is one of the major fruits of China; Hebei Province is the top producer of it in China. The pear juice industries produce tons of pear pomace as a by-product, which is a rich source of dietary fiber (1, 2); the dietary fiber is regarded as the “seventh major nutrient.” It mainly contains skin, flesh, and core; the total amount accounts for 40–50% of the fresh weight. According to the definition of the World Health Organization (3) and the International Codex Alimentarius Commission (3), dietary fiber contains all carbohydrate polymers of three or more monomers that cannot be digested as well as absorbed by human but can be broken down in the intestine by the gut microbiota (4). Based on solubility, dietary fibers can be divided into insoluble dietary fibers (IDF) and soluble dietary fiber (SDF). IDF can increase fecal bulk and decrease intestinal transit, but they are either slowly digested or not digested at all by gut microbiota. Contrary to this, SDF can be readily and quickly metabolized by gut microbiota, thus, significantly influences their abundance and diversity (5). SDF is made up of a variety of active substances with various structures, primarily oligosaccharides, resistant starch, and viscous dietary fiber with a high molecular weight (3), which are easily and quickly metabolized by the gut microbiota into monosaccharides or oligosaccharides after entering the colon and thus absorbed and utilized by the body in a specific way (6).

The gut microbiota plays an important role in maintaining health by regulating the host's immune system and metabolism (7). Gut microbiota dysbiosis is linked to the onset of obesity like metabolic illness, which is linked to the predominance of specific bacterial groups in the human gut (8, 9). Obesity is one of the most serious health and socioeconomic issues that human face in the twenty-first century. It is caused by a chronic imbalance between food intake and energy expenditure, which leads to excessive fat deposition (10). Obesity can induce additional metabolic syndromes (11), such as non-alcoholic fatty liver disease (NAFLD), insulin resistance (IR), and type 2 diabetes mellitus (T2DM) (12). Obesity can be prevented and its consequences can be reduced by reshaping the human gut microbiota by selectively manipulating the bacteria that predominate in fat people's stomachs (13). Obesity patients have distinct gut microbiota-derived metabolites; therefore, metabolite intervention can be a great target for treating obesity (14).

SDF intake can regulate the gut microbiota and can prevent obesity by improving energy homeostasis through the regulation of appetite and energy expenditure (15), by reducing white fat (4), and by improving glucose tolerance (16). SDF extracted from edible plants is a natural product that is of great interest because of its good functional activity and high safety profile (3). For instance, SDF extracted from walnut powder was shown to effectively reverse the perturbed gut microbiota and lipid metabolism as a result of HFD in mice (17). DF extracted from *Saccharina japonica* contains a high proportion of SDF, which can effectively improve metabolic disorders and gut microbiota dysbiosis in HFD-fed mice (18). A study with grapefruit peel total dietary fiber (TDF), SDF, and IDF showed an abundance and diversity of gut microbiota, confirming that SDF played a relatively significant role in modulating the gut microbiota in hypoglycemia (19). Obesity women blueberry soluble fiber supplementation may prevent excessive gestational weight gain and improve glycemic control and inflammation (20); Combined soluble fiber (CSF) supplementation decreased energy intake, promoted weight and fat loss, and augmented insulin sensitivity (21). Dietary fiber intake in children have negative effects on overweight and hypercholesterolemia (22). These research outcomes illustrated a theoretical foundation for preventing metabolic illnesses by changing the structural composition or metabolic activity of the gut microbiota through nutritional/dietary treatments (23, 24).

Pear pomace soluble dietary fiber (PP-SDF) functional activity to prevent obesity by regulating the gut microbiota has not been explored properly yet. The benefits pear, simmered with clove in treating nausea have been documented in the Compendium of Materia Medica, although the mechanism of action remains unknown. This study, for the first time, has reported the use of pear pomace soluble dietary fiber as an intervention strategy to target the gut microbiota to treat metabolic abnormalities in obese mice and explored the mechanism of action. We used high-throughput sequencing and untargeted metabolomics to reveal the role of PP-SDF in attenuating obesity through gut microbiota intervention in mice.

## Materials and methods

### Preparation of SDFs from pear pomace

Fresh ripe pears (*Pyrus bretschneideri*.) were picked from Zhao County, Hebei Province, China to obtain SDF. They were cut and homogenized by tissue homogenizer (JJ-2, Langbo Instrument Manufacturing Co., Jiangsu, China) after being soaked and washed with purified water and filtered through eight layers of gauze. Then the wet filter residue obtained by filtration is dried at 80°C. The products were grounded with a mortar to a finepaste to pass through a 60-mesh sieve. This experiment was modified based on the experiments of others (25). The probiotic strains used in this experiment were *Lactiplantibacillus plantarum Lp*-4 and *Lactobacillus acidophilus CH*-2 at 1:1 ratio. The cultures include pear pomace 30 g, milk powder 0.6 g, sugar 0.45 g, and water 210 mL. After sterilization, the cultures were incubated at 37°C for 24 h with continuous shaking at 200 rpm. At the end of fermentation, the supernatant was obtained after centrifugation at 4,600 × *g* for 25 min and then 95% ethanol was added to the supernatant for 24 h. The sediment was filtered and lyophilized to obtain PP-SDF.

### Experimental animal design and treatment

Experimentally naive 6-week-old SPF-grade C57BL/6J male mice were purchased from Beijing Spelford Biotechnology Co. (Beijing, China). The animal experiments in this study were approved and conducted under the guidelines of the Laboratory Animal Ethics Committee of Chenguang Biotechnology Group Co. (ZY-LL-W21002). The feed was divided into basic feed, control feed (10% of fat energy, No. D12450B), and high-fat feed (60% of fat energy, No. D12492); all of which were purchased from Beijing Spelford Biotechnology Co. The animals were housed in a cage (Five mice per cage) and kept in an air-conditioned room at 23 ± 1°C and 75–85% relative humidity with a 12 h light/12 h darkness cycle for 1 week before the experiment for adaptability.

After a week of adaptive feeding (Supplementary Figure 1), the mice were randomly divided into five groups (*n* = 16 each), and fed with a normal chow diet containing 10% fat (NC group, *n* = 16) or a high-fat diet containing 60% fat (other groups, *n* = 64) for 12 weeks. (1) Normal control group (NC): oral administration with an equivalent volume of 0.9% physiological saline. (2) high-fat-diet induced group (HFD): oral administration with an equivalent volume of 0.9% physiological saline. (3) low-dose soluble dietary fiber group (LSDF): oral administration with an equivalent volume of SDF (1 g/kg·BW), (4) medium-dose soluble dietary fiber group (MSDF): oral administration with an equivalent volume of SDF (3 g/kg·BW), (5) high-dose soluble dietary fiber group (HSDF): oral administration with an equivalent volume of SDF (5 g/kg·BW).

The daily oral administration of PP-SDF or physiological saline was 0.2 mL/kg for 12 weeks and body weight was measured weekly. At the end of the last week of the experiment, all mice were subjected to glucose tolerance test after 8 h of fasting. On the day before the experiment and execution, after 8 h of fasting, stool samples were collected and stored as week 0 or week 12 at −80°C until analysis. At the end of the experiment, after fasting overnight, blood was collected from the orbital sinus or plexus of the mice, and body length was measured. The liver and total adipose tissues (Epididymal or parametrial, perirenal, mesenteric, and inguinal subcutaneous depots) were weighted. The liver and epididymal fat were sectioned and all other tissues and organs were stored at −80°C until analysis.

Plasma biochemistry analyses: After blood collection, the serum was obtained, following centrifugation at 4°C for 10 min at 1917 g. The concentrations of triglyceride (TG), total cholesterol (TC), high-density lipoprotein (HDL) and low-density lipoprotein (LDL) in mice serum were measured, following the instructions of the corresponding kit (Jiancheng Biological Engineering Institute, Nanjing, China).

Measurement of triglyceride levels in the liver: A section of liver (0.1 g) was obtained and 0.9 mL phosphate buffer (pH = 7.2) was added for homogenization at low-temperature. The homogenized tissues were centrifuged at 4°C and 2,504 g for 10 min and the supernatant was collected. The triglyceride level was detected using the commercial kit (Jiancheng Biological Engineering Institute, Nanjing, China).

Oral glucose tolerance tests (OGTTs): OGTT was performed before the end of the experiment. The mice were fasted overnight and their weights were measured. The mice were given an intragastric injection of 2 g/kg glucose (100 mg/mL). The blood samples from the mice were collected from the tail vein and the blood glucose levels were measured by a Sinocare Glucose Meter (Company name of the Glucose Meter) at 0, 30, 60, 90 and 120 min.

### High-throughput sequencing and analysis of 16S ribosomal RNA gene in mice feces

The gut microbial genome was extracted from feces, using the OMG-soil Extraction Kit (Shanghai Meiji Biopharmaceutical Technology Co., Ltd., Shanghai, China). PCR amplification of the V3–V4 variable region of the 16S rRNA gene, using primers 338F (5′-ACTCCTACGGGAGGCAGCAG-3′) and 806R (5′-GGACTACHVGGGTWTCTAAT-3′). Sequencing was performed using Illumina's Miseq PE300 platform (Shanghai Meiji Biomedical Technology Co., Ltd.). The raw sequences were quality-controlled using FAST software; spliced using FLASH software; and the qualified reads were clustered to generate OTUs at the 97% similarity level, using UPARSE software. The chimera sequences were removed using RESEARCH software. Each sequence was annotated for species classification using the RDP classifier, compared to the Silva 16S rRNA database (Version 138) and a comparison threshold of 70% was set.

### Ultra-performance liquid chromatography/tandem mass spectrometry analysis of metabolites

Fifty mg solid sample was accurately weighed to extract the metabolites using a 400 μL methanol: water (4:1, v/v) solution. The mixture was allowed to settle at −20°C and treated by High throughput tissue crusher Wonbio-96c (Shanghai wanbo biotechnology Co., Ltd., Shanghai, China) at 50 Hz for 6 min. Then, it was vortexed for 30 s, followed by ultrasonication at 40 kHz for 30 min at 5°C. The samples were placed at −20°C for 30 min to precipitate proteins. After centrifugation at 13,000 × g at 4°C for 15 min, the supernatant was carefully transferred to sample vials for LC-MS/MS analysis. Chromatographic separation of the metabolites was performed on a ExionLCTMAD system (AB Sciex, USA), equipped with an ACQUITY UPLC HSS T3 (100 mm × 2.1 mm I.d., 1.8 μm; Waters, Milford, USA). The sample injection volume was 20 μL and the flow rate was set to 0.4 mL/min. The column temperature was maintained at 40°C. During the period of analysis, all these samples were stored at 4°C.

After UPLC-TOF/MS analyses, the raw data were imported into the Progenesis QI 2.3 (Waters Corporation, Milford, USA) for peak detection and alignment. The pre-processing results generated a data matrix that consisted of the retention time (RT), mass-to-charge ratio (*m/z*) values, and peak intensity. The software was then used to search the library for characteristic peaks and to match MS and MS/MS mass spectra with the metabolic database as the Human metabolome database (HMDB) (http://www.hmdb.ca/) and Metlin database (https://metlin.scripps.edu/), with the MS mass error set to <10 ppm. The pre-processed data were uploaded on the Megabio Cloud Platform (https://cloud.majorbio.com) for data analysis.

### Statistical analyses

All results were presented as mean ± standard error (SE). The difference between data were analyzed using one-way ANOVA, followed by Duncan's multiple-range test. Statistical significance was considered at *p* < 0.05.

## Results

### Effects of PP-SDF on obese mice fed with a high-fat diet

**Figure 1:**
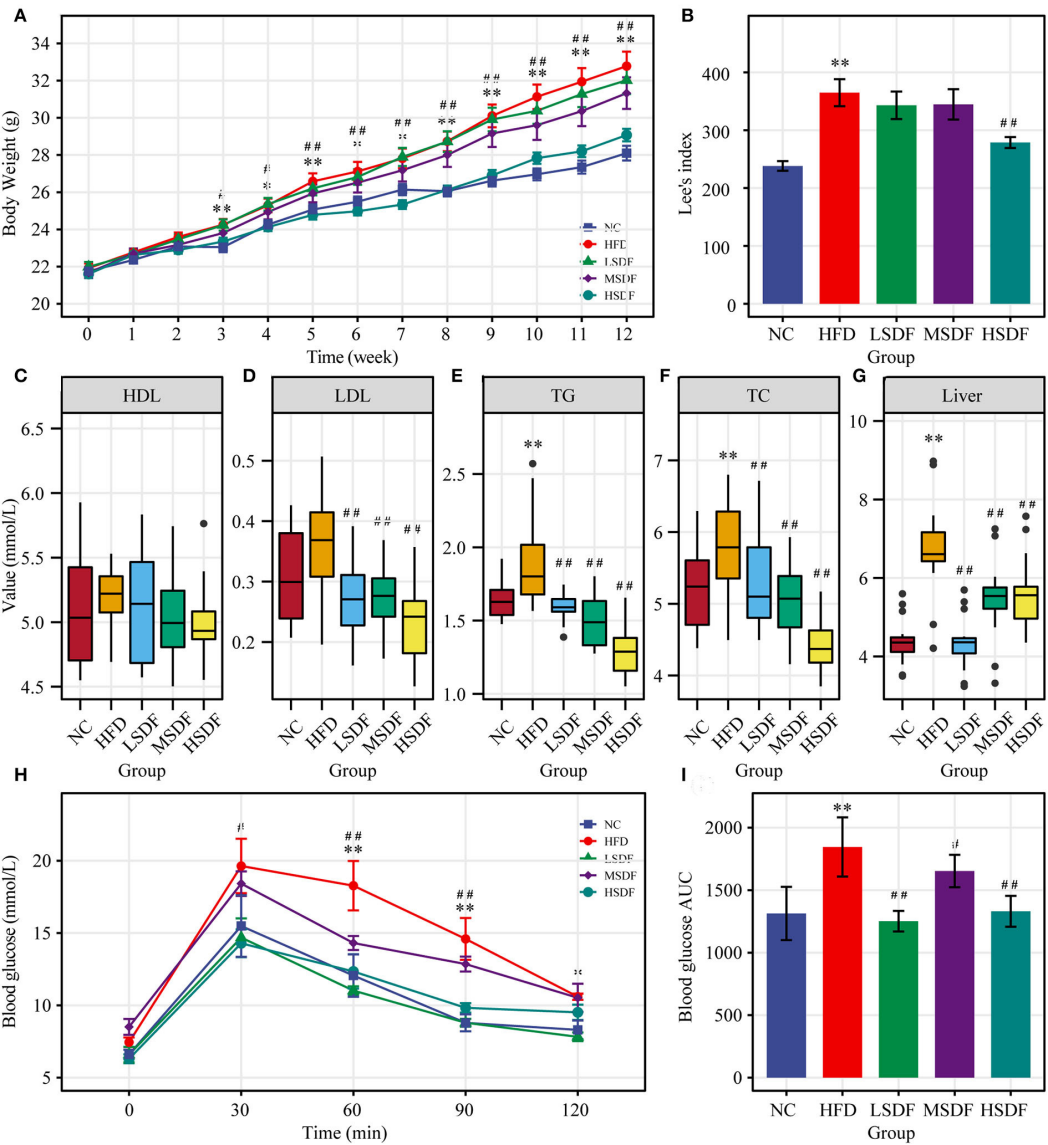
Effect of PP-SDF administration on metabolic disorders in obese mice induced by a high-fat diet. **(A)** Time-course of body weight. **(B)** Obesity index of different groups. **(C)** Serum high-density lipoprotein (HDL). **(D)** Serum low-density lipoprotein (LDL). **(E)** Serum triglyceride (TG). **(F)** Serum total cholesterol (TC). **(G)** Liver triglycerides. **(H)** Curve of OGTT. **(I)** ATU of OGTT. NC: 10% fed on a low-fat diet, oral administration with an equivalent volume of 0.9% physiological saline; HFD: 60% fed on a high-fat diet, oral administration with an equivalent volume of 0.9% physiological saline; LDF, MSDF, and HSDF: 60% fed on a high-fat diet, different concentrations of SDF solution were gavaged: 1g/kg·BW, 3 g/kg·BW, and 5 g/kg·BW, respectively. Values were presented as mean ± SE (*n* = 16). The high-fat group was compared with the blank control group, which was represented by *. The dose group was compared with the high-fat group, denoted by ^*##*, *^p < 0.05; ^*##*, **^p < 0.01.

After 12 weeks of feeding, the body weight of the HFD-fed mice group was significantly greater than body weight of the mice in the NC group (*p* < 0.01) (Figure 1A), indicating that the HFD-induced obesity mice model was successfully established (Figure 1B). Compared with the HFD-fed mice, the body weight of PP-SDF-fed mice of all the three groups was less but a significant difference (*p* < 0.01) was noted only in HSDF-fed mice. PP-SDF markedly reduced the accumulation of white fat in HFD fed- obese mice, compared to the HFD group mice (*p* < 0.05). The fat weights measured in five different anatomical sites, including subcutaneous, epididymal, perirenal, inguinal, and mesenteric (Table 1) showed a significantly higher values in the HFD group, when compared with the NC group (*p* < 0.05).

**Table 1:** Effects of the PP-SDF on the relative masses of adipose tissue of mice.

**Adipose**	**NC**	**HFD**	**LSDF**	**MSDF**	**HSDF**
Subcutaneous fat	0.29 ± 0.01^d^	0.71 ± 0.03^a^	0.53 ± 0.03^b^	0.49 ± 0.02^bc^	0.43 ± 0.03^c^
Epididymal fat	0.75 ± 0.10^d^	1.63 ± 0.15^a^	1.09 ± 0.23^b^	0.94 ± 0.17^b^	1.03 ± 0.15^bc^
Perirenal fat	0.32 ± 0.05^c^	0.51 ± 0.14^a^	0.28 ± 0.07^b^	0.31 ± 0.09^b^	0.28 ± 0.09^b^
Mesenteric fat	0.02 ± 0.01^b^	0.05 ± 0.01^a^	0.03 ± 0.01^b^	0.03 ± 0.01^b^	0.03 ± 0.01^b^
Inguinal Fat	0.23 ± 0.08^c^	0.60 ± 0.11^a^	0.38 ± 0.11^b^	0.37 ± 0.08^b^	0.36 ± 0.10^b^

The data within the same row followed by different superscript letters differ significantly (*p* < 0.05). Values were presented as mean ± SE. NC: 10% fed on a low-fat diet, oral administration with an equivalent volume of 0.9% physiological saline; HFD: 60% fed on a high-fat diet, oral administration with an equivalent volume of 0.9% physiological saline; LDF, MSDF, and HSDF: 60% fed on a high-fat diet; different concentrations of SDF solution were gavaged, 1, 3, and 5 g/kg·BW, respectively.

When compared to the NC group, HFD resulted a significant increase in the serum concentrations of TG and TC levels (*p* < 0.05). In addition, HFD resulted increase in the serum concentrations of LDL levels (Figures 1D–F), but not significant, while HDL levels were not significant in these five groups (Figure 1C). Compared to the HFD group, serum levels of TG, TC and LDL were significantly lower in all three PP-SDF groups (Figures 1D–F) (*p* < 0.01). PP-SDF administration notably decreased the serum levels of TC, TG, and LDL, most probably induced by HFD, but had no modulating effect on serum HDL (Figure 1C). Compared to the NC group, liver TG levels were also found significantly higher in the HFD group (*p* < 0.01) (Figure 1G). PP-SDF intake significantly reduced the TG levels in liver due to a high-fat diet (*p* < 0.01). Unlike serum TG levels, liver TG levels increased with increasing PP-SDF concentrations.

The OGTT results showed that the blood glucose level of the HFD-fed group mice at 30, 60, 90, and 120 min was higher than that in other groups, while the glucose levels of all group mice reached a peak value at 30 min after the administration of glucose and gradually decreased to the pre-prandial levels (Figure 1H). The corresponding area under curve (AUC) values further confirmed that the LSDF, MSDF and HSDF notably ameliorated the impaired glucose tolerance in HFD-fed group mice (Figure 1I). Thus, the PP-SDF reduced the blood glucose level. Among them, the LSDF and HSDF groups were more significant (*p* < 0.01).

### Effect of SDFs on gut microbiota

**Figure 2:**
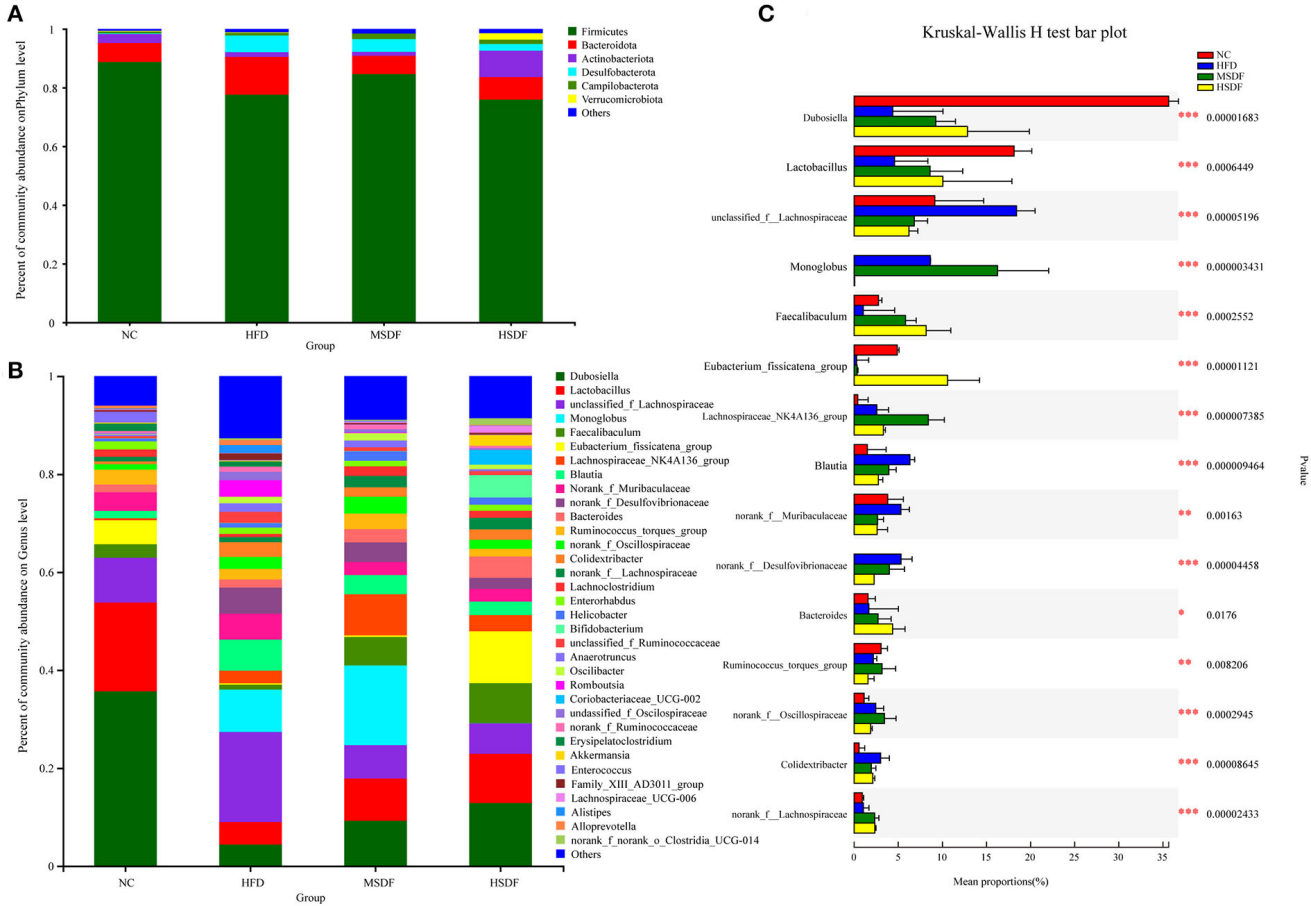
Effect of PP-SDF administration on the composition of the gut microbiota in phylum and genus levels. **(A)** At the 12-week phylum level. **(B)** At the 12-week genus level. **(C)** The difference between groups was tested at the genus level. Values were presented as mean ± SD (*n* = 8). The variability between groups was represented by *.

Physiological and biochemical indexes showed that LSDF-fed mice did not have any apparent changes in the tested parameters, and thus we excluded this group from further trials thus two intervention groups, MSDF and HSDF, were selected for the follow-up experiment. At the beginning of the trial at 0-week (Supplementary Figure 2A), a total of three phyla were identified in sequencing analysis, mainly including Firmicutes, Bacteroidetes and Campilobacterota, and no differences were observed among 4 groups (NC, HFD, MSDF, and HSDF). Verrucomicrobia, Actinobacteria, and Desulfobacterota appeared in week 12, while the level of Firmicutes increased significantly, and the level of Bacteroidetes decreased significantly in 4 groups (Figure 2). Compared to the NC group, the Desulfobacterota levels seemed to increase, whereas Actinobacteria levels appeared to reduce in the HFD-fed mice (*p* < 0.01). Compared to the HFD-fed group mice, the intake of PP-SDF led to an increase in the level of Actinobacteria and Verrucomicrobia (*p* < 0.01), and a decrease in the level of Desulfobacterota (*p* < 0.01).

Bacterial community dynamics were also evaluated, based on the changes at genus level. At 0-week, no differences were observed in the 4 groups (Supplementary Figure 2B). A total of 34 bacterial genera were identified by high-throughput sequence (Figure 2B). In the NC group, *Dubosiella* was found as the predominant genus. Compared with the NC group, the abundance of *unclassified_f_lachnospiraceae, norank_f_Desulfovibrionaceae, Alistipes, Colidextribacter, unclassified_ f_Ruminococcaceae, Romboutsia, unclassifified_ f_Oscillospiraceae, Alloprevotella, Anaerotrumcus*, and *Family_*XIII_AD301_group increased in the HFD group, while *Dubosiella, Lactobacillus, Lachnospiraceae_*NK4A136_group, *norank_f_ Lachnospiraceae, Lachnoclostridium*, and *Eubacterium_* fissicatena_group decreased. Compared with the HFD-fed mice, both MSDF and HSDF group mice showed a similar trend of the gut microbiota changes, as the abundance of *Akkermansia, Coriobacteriaceae_*UCG-002*, Bifidobacterium, Faecalibaculum, Lachnospiraceae_*UCG-006, and *Dubosiella* was observed in mice belonging to both groups. The significance of the difference between groups was tested at the genus level (Figure 2C). Compared to the four groups, there were 15 species belonging to different genera, including *Dubosiella, Faecalibaculum, Lactobacillus, unclassified_f_Lachnospiraceae, Monoglobus, norank_f_Desulfovibrionaceae, Eubacterium_fissicatena_group, Lachnospiraceae_NK4A136_group, Blautia, norank_f__Muribaculaceae, Bacteroides, Ruminococcus_torques_group, norank_f__Oscillospiraceae, norank_f__Lachnospiraceae*, and *Colidextribacter*. Mice fed with HFD showed a significant reduction in the abundance of *Dubosiella, Faecalibaculum* and *Lactobacillus*, whereas, an increase in the abundance of *unclassified_f_Lachnospiraceae, norank_f_Desulfovibrionaceae*, and *Colidextribacter* was observed. However, in PP-SDF-fed mice groups, an increase in the level of those genera was observed (which were reduced as a result of HFD) and the microbiota was more balanced.

**Figure 3:**
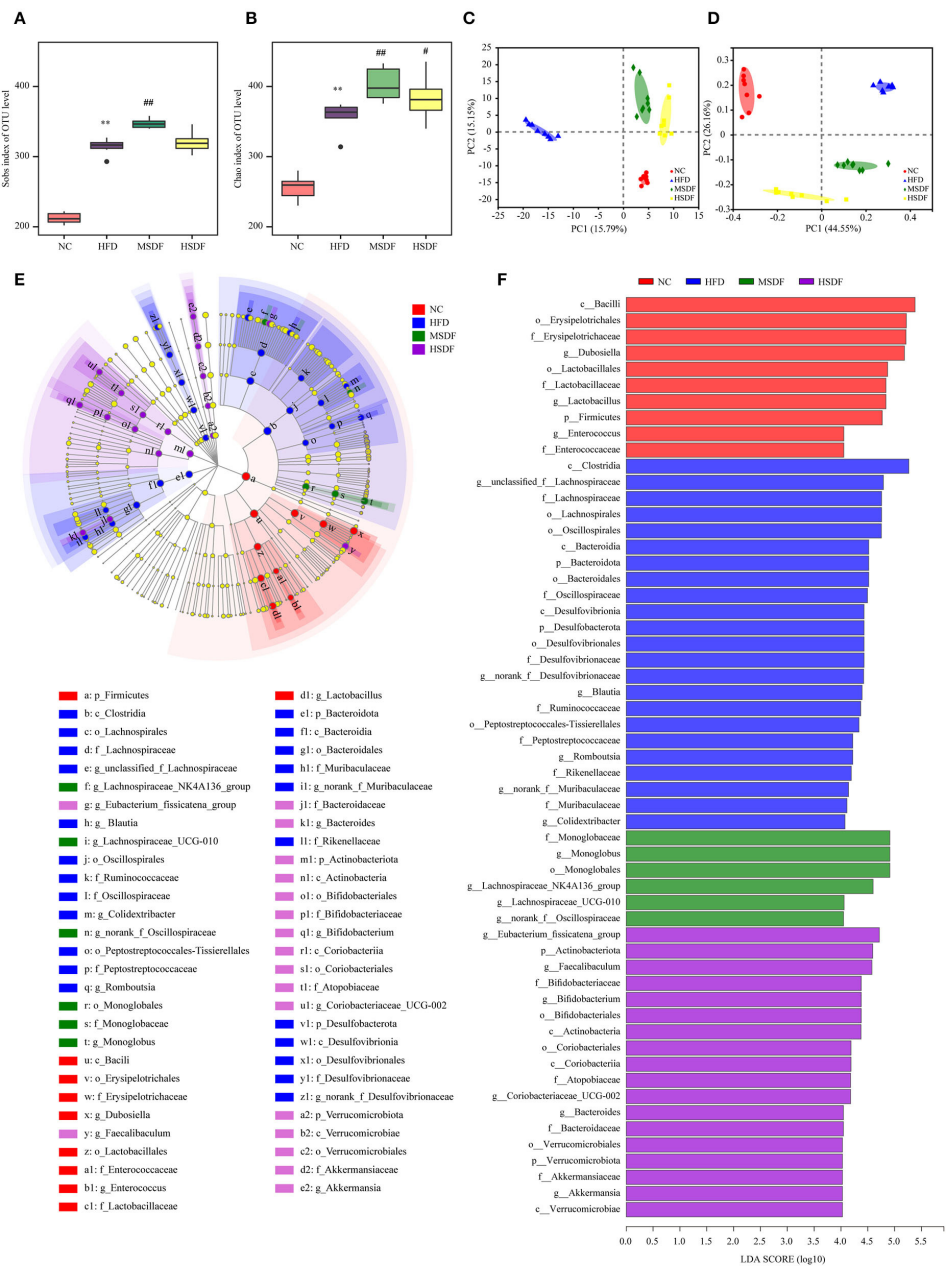
Effect of PP-SDF administration on the structure of the gut microbiota. Alpha diversity was evaluated by Chao and Sobs index. **(A)** Sobs index at 12 weeks. **(B)** Chao index at 12 weeks. **(C)** PCA of bacterial community composition at 12 weeks. **(D)** PCoA plot of weighted UniFrac distance at 12 weeks. **(E)** Cladogram generated from LEfSe analysis. **(F)** Linear discriminant analysis (LDA) scores. NC: 10% fed on a low-fat diet, oral administration with an equivalent volume of 0.9% physiological saline; HFD: 60% fed on a high-fat diet, oral administration with an equivalent volume of 0.9% physiological saline; MSDF, and HSDF; 60% fed on a high-fat diet. Different concentrations of SDF solution were gavaged, 3 g/kg·BW, and 5 g/kg·BW, respectively. Values were presented as mean ± SD (*n* = 8). The high-fat group was compared with the blank control group, which was represented by *. The dose group was compared with the high-fat group, denoted by ^#^ and *. *P* < 0.05^##^ and **p < 0.01.

Sobs and Chao indexes were used to evaluate the α diversity of gut microbiota. At the 0-week, no differences were observed in the 4 groups (Supplementary Figure 3). While at the 12 weeks, gut microbiota α diversity was improved in PP-SDF groups and it was significant increase in the level of diversity from (in the HFD-fed) in the MSDF-fed mice group (Figures 3A,B). Changes in the overall structure of the gut microbiota were studied by PCA and PCoA. Before the feeding test at the 0 week (Supplementary Figure 3), all samples clustered together, suggesting no differences were observed in the 4 groups. While after 12 weeks, significant differences were found in the 4 groups. The HFD group and the PP-SDF groups were divided into two clusters (Figure 3C). Furthermore, PC2, which explained 26.16% of the total variance, showed that PP-SDF, shifted the gut microbiota composition of mice fed HFD toward the gut microbiota profile of the NC group mice (Figure 3D).

The LEfSe analysis of colonic microbiota is shown in Figures 3E,F. At the class level, *Clostridia* were enriched in the HFD group, compared to any other group. At the genus level, *Blautia, Romboutsia*, and *Colidextribacter* were enriched in the HFD group, whereas *Dubosiella, Lactobacillus*, and *Enterococcus* enriched in the NC group. *Monoglobus, Lachnospiraceae_*NK4A136_group, *Lachnospiraceae_*UCG-010, and *norank_f__Oscillospiraceae* were enriched in the MSDF group. *Eubacterium*_fissicatena_group, *Faecalibaculum, Bifidobacterium, Coriobacteriaceae*_UCG-002, *Bacteroides*, and *Akkermansia* were enriched in the HSDF group.

### Effect of SDF on metabolites of colon content

To demonstrate the effect of PP-SDF on the host metabolism, a metabolome analysis of colonic contents was performed. After a series of pretreatments, including normalization and dealing with missing values, a total of 7,591 and 7,120 different kinds of mass spectrum peaks were detected in colon contents samples in positive and negative ion modes and the number of annotated substances was 713 through primary and secondary mass spectrum data searching (Commercial library majorbio in -home database, Metlin, HMDB etc.).

**Figure 4:**
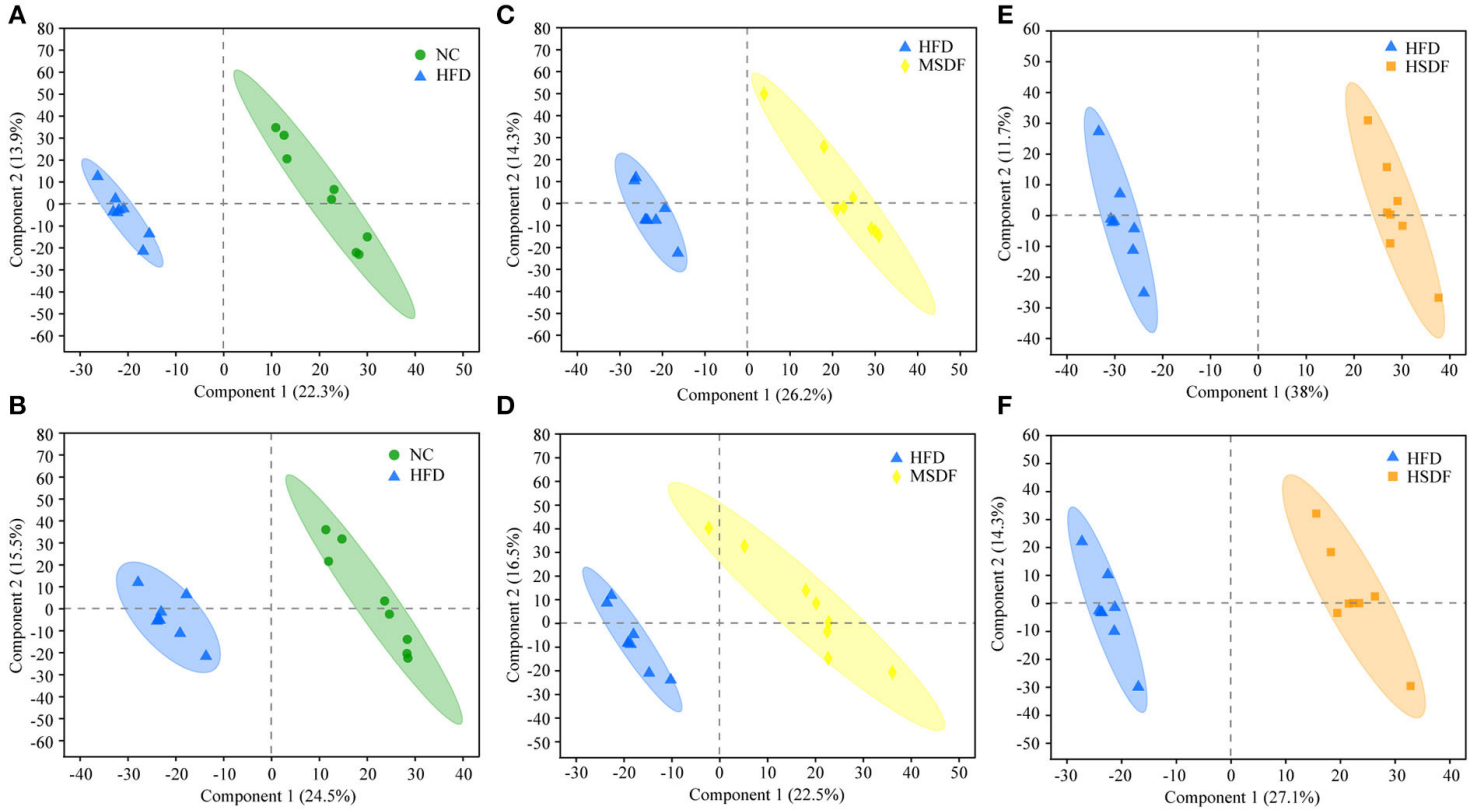
PLS-DA score plots. **(A)** PLS-DA model plot for the comparison group HFD vs. NC, positive ion mode, R^2^X = 0.362, R^2^Y = 0.995, *Q*^2^ = 0.934; **(B)** PLS-DA model plot for the comparison group HFD vs. NC, negative ion mode, R^2^X = 0.472, R^2^Y = 0.998, *Q*^2^ = 0.922; **(C)** PLS-DA model plot for the comparison group MSDF vs. HFD, positive ion mode, R^2^X = 0.405, R^2^Y = 0.991, *Q*^2^ = 0.943; **(D)** PLS-DA model plot for the comparison group MSDF vs. HFD, negative ion mode, R^2^X = 0.541, R^2^Y = 0.993, *Q*^2^ =0.913; **(E)** PLS-DA model plot for the comparison group HSDF vs. HFD, positive ion mode, R^2^X = 0.497, R^2^Y = 0.996, *Q*^2^ = 0.977; and **(F)** PLS-DA model plot for the comparison group HSDF vs. HFD, negative ion mode, R^2^X = 0.414, R^2^Y = 0.992, *Q*^2^ = 0.937. Values were presented as mean ± SD (*n* = 8).

Differential changes in the metabolic profile of the mice belonging to NC, HFD, MSDF, and HSDF were analyzed by PCA. However, it could not distinguish the four groups, while further multivariate statistical analysis was necessary to obtain more detailed information about the metabolic alterations after modeling (Supplementary Figure 4). PLS-DA distinguished HFD and NC groups into distinct clusters, based on differences in metabolites resulting from different dietary patterns (Figures 4A,B). Meanwhile, the PP-SDF groups and HFD group into distinct clusters, based on differences in metabolites resulting from PP-SDF intervention (Figures 4C–F). The quality metrics (R^2^X, R^2^Y, and Q^2^) in the PLS-DA model clearly showed that the metabolite composition of the colonic contents from mice of different groups were variable. The R^2^Y values of the model in both positive and negative ion modes were >0.99, indicating that the model had good adaptability to the experimental data. The *Q*^2^ values of 0.934 for the positive ion mode and 0.922 for the negative ion mode reflected that the PLS-DA model showed good predictability for the data sets.

Potential biomarkers were selected, based on the PLS-DA model by variable importance in the projection score (Fold change 1.5; *p* < 0.05; VIP >1). Compared with the NC group, all 5 metabolites were reduced in the HFD group. When metabolites of the MSDF group were compared with the HFD group, 3 metabolites were found in the MSDF group, where [3-(2-hydroxy-4-methoxyphenyl) prop-2-en-1-yl] oxy sulfonic acid was reduced and PE[18:4(6Z,9Z,12Z,15Z)/20:5(5Z,8Z,11Z,14Z,17Z)] and isobutyryl carnitine were increased. Compared with the HFD group, 8 metabolites were found in the HSDF group; 6 metabolites were increased; and 2 metabolites were decreased. Two of the 8 metabolites appeared and decreased in both HSDF and MSDF groups, which included PE[18:4(6Z,9Z,12Z,15Z)/20:5(5Z,8Z,11Z,14Z,17Z)] and isobutyryl carnitine.

**Figure 5:**
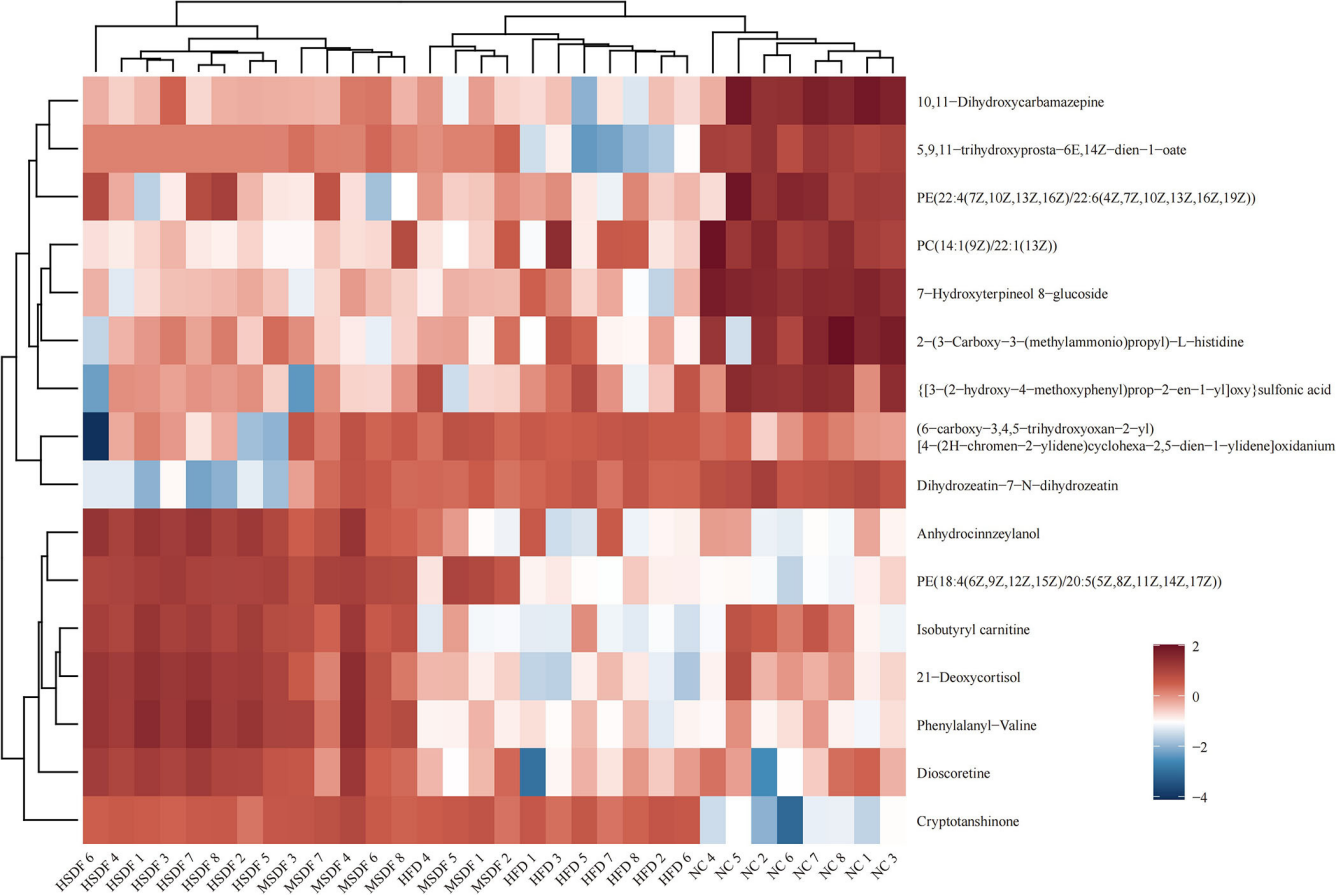
Heat map of metabolite clustering analysis of colonic contents (*n* = 8). Red indicates up-regulation and blue down-regulation. Columns and rows represent colonic content samples and metabolites, respectively.

Heat map showing the results of the cluster analysis of metabolite of 32 colonic contents are shown in Figure 5. Compared with the NC group, 8 metabolites were down-regulated in the HFD group, including PC[14:1 (9Z)/22:1 (13Z)], 2-[3-Carboxy-3-(methylammonio) propyl]-L-histidine, 7-Hydroxyterpineol 8-glucoside, [3-(2-hydroxy-4-methoxyphenyl) prop-2-en-1-yl]oxy sulfonic acid, PE[22:4(7Z,10Z,13Z,16Z)/22:6 (4Z,7Z,10Z,13Z,16Z,19Z)], 5,9,11-trihydroxyprosta-6E,14Z-dien-1-oate, 10,11-Dihydroxycarbamazepine, and isobutyryl carnitine. Compared with HFD group, PP-SDF intake caused anhydrocinnzeylanol, PE[18:4(6Z,9Z,12Z,15Z)/20:5 (5Z,8Z,11Z,14Z,17Z)], isobutyryl carnitine, 21-deoxycortisol, phenylalanyl-valine, and dioscoretine to increase. Among them, the upregulation trend was more significant in the HSDF group, compared with the MSDF group.

**Figure 6:**
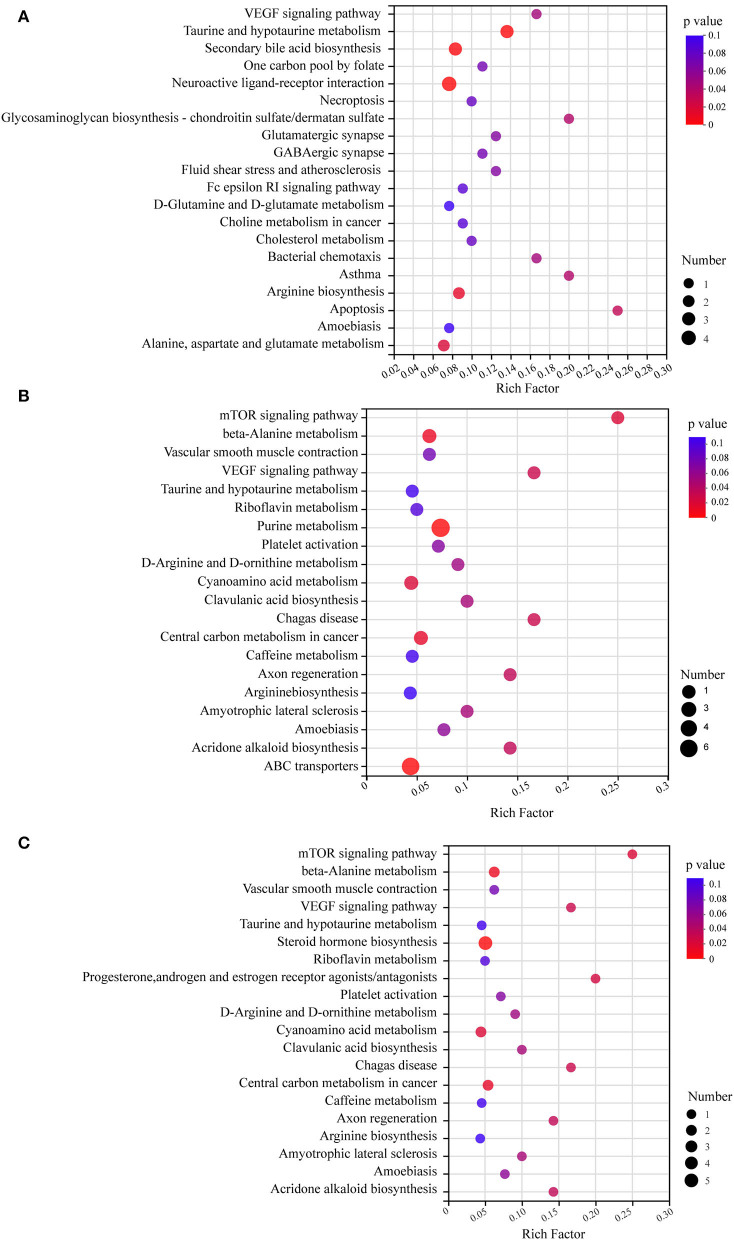
KEGG pathway enrichment analysis bubble diagram. The top 20 metabolic pathways were selected (*n* = 8). **(A)** HFD vs. NC. **(B)** MSDF vs. HFD. **(C)** HSDF vs. HFD. The horizontal coordinate is the rich factor, calculated as several differential metabolites/total metabolites in each pathway. The size of the bubbles in the graph represents the number of metabolites enriched to the metabolic set in that pathway and the color of the bubbles indicates the size of different enrichment significance *p*-values.

KEGG pathway enrichment analysis revealed pathways associated with differential metabolites (Figure 6). All the differential metabolites were a part of 79 metabolic pathways in the HFD-fed mice group, compared with the NC group and the top 20 metabolic pathways screened by degree of enrichment are shown in Figure 6A based on the rich factor, including the VEGF signaling pathway, One carbon pool by folate, Necroptosis, Glycosaminoglycan biosynthesis—chondroitin sulfate/dermatan sulfate, Glutamatergic synapse, GABAergic synapse, Fluid shear stress and atherosclerosis, Fc epsilon RI signaling pathway, D-Glutamine and D-glutamate metabolism, Choline metabolism in cancer, Cholesterol metabolism, Bacterial chemotaxis, Asthma, Apoptosis, Amoebiasis, Arginine biosynthesis, Alanine, aspartate and glutamate metabolism, Taurine and hypotaurine metabolism, Secondary bile acid biosynthesis, and Neuroactive ligand-receptor interaction.

Four metabolic pathways were identical in the top 20 metabolic pathways of MSDF and HSDF groups (Figures 6B,C), compared with the HFD group, including mTOR signaling pathway, beta-Alanine metabolism, Vascular smooth muscle contraction, VEGF signaling pathway, Taurine and hypotaurine metabolism, Riboflavin metabolism, Platelet activation, D-Arginine and D-ornithine metabolism, Cyanoamino acid metabolism, Clavulanic acid biosynthesis, Chagas disease, Central carbon metabolism in cancer, Caffeine metabolism, Axon regeneration, Arginine biosynthesis, Amyotrophic lateral sclerosis, Amoebiasis, and Acridone alkaloid biosynthesis.

### Correlation of differential metabolites and gut microbiota after PP-SDF intervention

**Figure 7:**
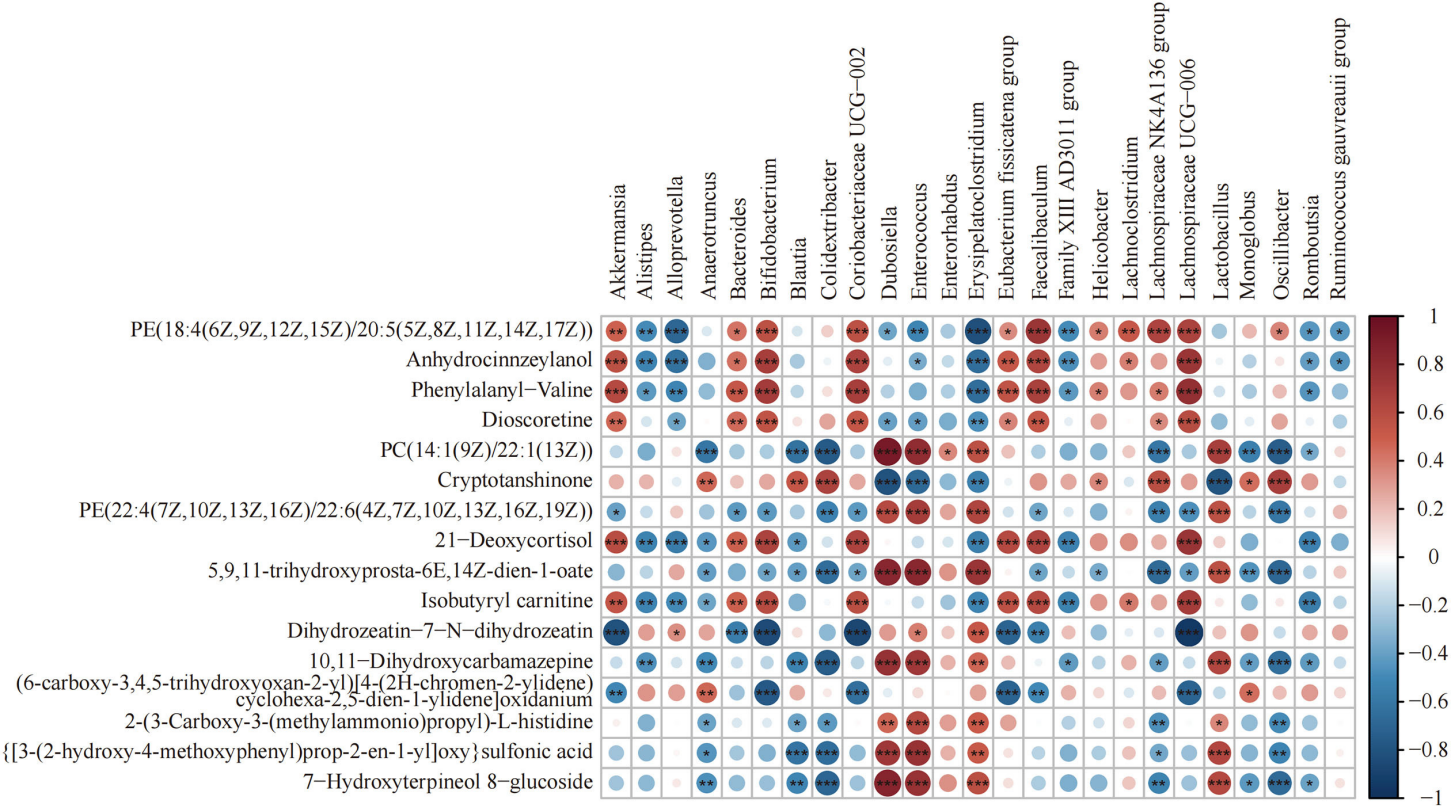
Heat map of the correlation between differential bacteria of the gut microbiota and differential metabolites (*n* = 8). Differential genera and their abundance in the four groups of colonic contents samples were obtained by LEfSe analysis. Differential metabolites were obtained from the NC and HFD groups and the MSDF and HSDF groups for comparison. *p < 0.05; **p < 0.01; ***p < 0.001.

Heat map analysis of the correlation between the gut microbiota and metabolites is shown in Figure 7. *Lachnospiraceae_*UCG-006 and Anhydrocinnzeylanol, 21-Deoxycortisol, Dioscoretine, Isobutyryl carnitine, PE[18:4(6Z,9Z,12Z,15Z)/20:5 (5Z,8Z,11Z,14Z,17Z)], and Phenylalanyl-valine showed a strong correlation (*p* < 0.001; *r* > 0.5). *Coriobacteriaceae_*UCG-002*, Bacteroides, Faecalibaculum, Bifidobacterium*, and *Akkermansia* showed a positive correlation with the above metabolites. *Alistipes, Enterococcus, Erysipelatoclostridium*, and *Alloprevotella* showed a negative correlation with the above metabolites.

## Discussion

Lee index is a common indicator, which is used to evaluate the level of obesity. The results related to body weight, Lee index, and blood lipids showed that PP-SDF attenuated HFD-induced fat deposition, Lee index, and serum TG, LDL and TC. Body weight and white fat were all significantly higher in mice after 12 weeks on the HFD, which was consistent with earlier studies (26–29). White fat deposition is a major cause of dyslipidemia, which is characterized by elevated serum TG, LDL and TC levels, and reduced level of HDL (30). The PP-SDF intervention strategy in our trial led to a great reduction of serum TG, LDL and TC, and liver TG levels. Unlike other studies (31), we discovered no significant differences in HDL levels in the five groups in our investigation, and we concluded that PP-SDF ingestion had no influence on HDL levels. Obesity occurs when energy intake exceeds energy expenditure (32), thus leading to an abnormal accumulation of body fat (33). It has been demonstrated that SDF can improve HFD-induced disrupted energy homeostasis and this can be linked to its role in regulating the gut microbiota (3).

Body weight and blood lipid levels are closely related to the composition of the gut microbiota, which can be regulated by dietary intervention (34). Studies showed that dietary fiber, especially SDF have some effects on establishing the healthy gut microbiota by regulating the ratio of beneficial and the harmful bacteria (3). In this study, α-diversity was evaluated by Sobs and Chao indices, which showed a significantly higher bacterial community diversity and richness on the PP-SDF intervention, which was similar with the results of other studies (35). The ratio of Firmicutes to Bacteroidetes is linked to the organism's health, particularly in the setting of obesity (36). A previous study demonstrated a high Firmicutes to Bacteroidetes ratio in obesity (37). A high-fat diet reduces the Firmicutes to Bacteroidetes ratio (36). We discovered in this study that the PP-SDF intervention increased the Firmicutes to Bacteroidetes ratio, most probably because of a rise in the number of good bacteria in Firmicutes and Bacteroidetes. Firmicutes include *Lactobacillaceae* like probiotics; Bacteroidetes also include *Dubosiella* like beneficial bacteria. At the genus level, some harmful bacteria, associated with obesity were enriched in HFD-fed group; such bacteria included *Desulfovibrionaceae, Alistipes, Alloprevotella, Ruminococcaceae*, and *Anaerotruncus* (38). In this study, some beneficial bacterial, such as *Akkermansia, Faecalibaculum, Bifidobacterium, Lachnospiraceae_*UCG-006*, Coriobacteriaceae*, and *Dubosiella* were enriched in PP-SDF intervention group. Among them, *Akkermansia, Bifidobacterium, Faecalibaculum*, and *Lachnospiraceae_*UCG-006 are common probiotics that have been shown in several studies to reduce weight by strengthening the gut barrier and reducing inflammation (39). *Faecalibaculum* is capable of releasing short chain fatty acids (SCFAs), causing an increase in energy metabolism (40). An increase in the abundance of *Akkermansia* may, to some extent, alleviate metabolic disorders caused by HFD, including increased fat mass, insulin resistance, and other metabolic disorders (41, 42). *Lachnospiraceae*_UCG-006 belongs to a genus of the *Lachnospiraceae* family. Bacteria belonging the *Lachnospiraceae* family are core members of the gut microbiota (43), which colonize the intestinal lumen and start increasing from birth (44). *Lachnospiraceae_*UCG-006 was negatively associated with gut inflammation because of its ability to produce SCFAs (45). SCFAs maintained intestinal barrier function and reduced lipopolysaccharides content in blood, thereby helping to reduce systemic inflammation and reverse insulin resistance (45). In obese mice, increased levels of *Lachnospiraceae*_UCG-006 were found to regulate the gut microbiota and improve glucose metabolism disorders (46). *Dubosiella* can alleviate gut disorders caused by HFD but many studies have found members of this genera to be detrimental; so, further studies are needed to fully elucidate its role in the human gut (47). In summary, *Akkermansia, Lachnospiraceae*_UCG-006, *Faecalibaculum, Dubosiella*, and *Bifidobacterium* can reduce obesity due to high-fat diet by reducing intestinal inflammation. The composition of the gut microbiota is altered by dietary intervention and beneficial bacteria can be targeted through specific food ingredients/prebiotics. *Akkermansia and Lachnospiraceae_*UCG-006 (45) have shown to be regulated by SDF in this study as well as in previous studies (48–50).

Results of differential metabolites are shown in Table 2. Among them, isobutyryl carnitine (51, 52), dioscoretine (53–55), and phosphatidylethanolamine (PE) (56, 57) are closely related to lipid metabolism. Anhydrocinnzeylanol (A terpene lactone) has an important role in suppressing inflammatory responses (58). Inflammation is also closely related to lipid metabolism (59). It is therefore speculated that PE may be a potential target for the intervention of obesity induced by HFD.

**Table 2:** Identification of different metabolites from each group of NC, HFD, MSDF, and HSDF.

**Compared groups**	**Metabolite**	**Formula**	**VIP_PLS-DA**	**FC**	***m/z***
HFD vs. NC	10,11-Dihydroxycarbamazepine	C15H14N2O3	3.42	0.65	307.05
	2-(3-Carboxy-3-(methylammonio) propyl)-L-histidine	C11H19N4O4+	2.74	0.63	270.13
	7-Hydroxyterpineol 8-glucoside	C16H28O7	3.93	0.56	313.16
	PE[22:4(7Z,10Z,13Z,16Z)/22:6(4Z,7Z,10Z,13Z,16Z,19Z)]	C49H78NO8P	3.19	0.68	442.77
	Isobutyryl carnitine	C11H21NO4	3.25	0.37	232.15
MSDF vs. HFD	{[3-(2-hydroxy-4-methoxyphenyl) prop-2-en-1-yl]oxy}sulfonic acid	C10H12O6S	2.59	0.66	305.03
	PE[18:4(6Z,9Z,12Z,15Z)/20:5(5Z,8Z,11Z,14Z,17Z)]	C43H68NO8P	4.46	1.77	780.46
	Isobutyryl carnitine	C11H21NO4	3.48	3.18	232.15
HSDF vs. HFD	Dihydrozeatin-7-N-dihydrozeatin	C16H25N5O6	4.40	0.47	418.15
	(6-carboxy-3,4,5-trihydroxyoxan-2-yl)[4-(2H-chromen-2-ylidene)cyclohexa-2,5-dien-1-ylidene]oxidanium	C21H19O8+	2.63	0.69	380.09
	PE[18:4(6Z,9Z,12Z,15Z)/20:5(5Z,8Z,11Z,14Z,17Z)]	C43H68NO8P	3.69	1.84	780.46
	Isobutyryl carnitine	C11H21NO4	4.13	4.70	232.15
	Anhydrocinnzeylanol	C20H30O6	2.84	1.65	389.19
	Phenylalanyl-Valine	C14H20N2O3	3.35	1.79	247.14
	Dioscoretine	C13H23NO3	2.58	1.59	274.20
	21-Deoxycortisol	C21H30O4	2.95	1.65	385.17

Blue font: Metabolites that were down-regulated in the HFD group, compared to the NC group; red font: Metabolites that were up-regulated in the intervention group, compared to the HFD group. The metabolites in this box are the main substances intervened by pear pomace dietary fiber in this study and the metabolic pathways BACCs and lipid metabolism, in which they are located, are postulated as possible pathways of action for the effects of the interventions.

Regulation of the gut microbiota is accompanied by metabolic changes and many metabolites produced by gut microbes affect host metabolic phenotype (24). In addition to glycerophospholipids, fatty acids are also included in lipids. Dioscoretine is an unsaturated fatty acid metabolite that has been shown to act as a blood glucose suppressant to reduce high blood glucose in diabetes (53). It also improves glucose tolerance and increases serum insulin levels (55). In the present study, HFD reduced the level of dioscoretine in mice, whereas dioscoretine was maintained at a higher level in the HSDF group. It showed that the intake of PP-SDF could increase the level of glycerophospholipid and increase blood glucose levels that are disturbed as a result of HFD.

L-carnitine and acylcarnitines are closely related to lipid metabolism. L-carnitine made up of the amino acids, namely lysine and methionine that reduces body fat gain caused by the HFD (60) and improves insulin resistance (61). Isobutyryl carnitine belongs to the acylcarnitines of L-carnitine, which means that they may have a role in reducing blood glucose level and enhancing lipolysis in mice fed HFD. In addition, L-carnitine and acylcarnitines are established mitochondrial biomarkers, used to screen neonates for a series of genetic disorders (Inborn errors of metabolism), affecting fatty acid oxidation (62, 63). Isobutyryl carnitine, also known as isobutyryl-L-carnitine, is similar to L-carnitine in its function of transferring long-chain fatty acids from the cytoplasm to the mitochondrial matrix for β-oxidation and in way, it assists in the transport of short- and medium-chain fatty acids in the mitochondria (64, 65). Under the current study, which was similar to other studies (65), isobutyryl carnitine demonstrated a negative correlation with fat deposition and obesity as a result of HFD. Our data revealed the PP-SDF intervention effects for the levels of isobutyryl carnitine. This might be partly attributable to isobutyryl carnitine to enhance lipid metabolism. However, direct regulation of lipid metabolism by isobutyryl carnitine has been less studied. L-carnitine is a commonly used as an adjunct in some drugs used for weight loss (60). However, exogenous intake can cause many side effects, such as arrhythmia, loss of appetite and concentration, and even cardiac arrest and liver damage. In addition, another weight loss aid is peptide, which are very small active peptides that can prevent fat from being absorbed (66) and effectively reduce body fat in people while keeping bone density constant. They can also purify excess blood lipids and encourage the synthesis of muscle protein (67). In the present study, isobutyryl carnitine and phenylalanyl-valine was significantly upregulated by PP-SDF intervention and high safety profile.

In order to better obtain the interactions between resultant gut microbiota changes and produced metabolites by following different PP-SDF-based interventions, we performed association analysis of differential flora with differential metabolites. The findings revealed that genera like *Alistipes* enriched in the HFD group and had a strong negative correlation with both metabolites, namely isobutyryl-l-carnitine and dioscoretin, whereas genera like *Akkermansia* and *Lachnospiraceae*_UCG-006 enriched in the PP-SDF-based interventions and had a strong positive correlation with these two metabolites. Therefore, we conjecture that PP-SDF was able to regulate certain beneficial microbiota which was in line with the metabolites produced by the corresponding bacteria. This provides evidence that a significant increase in metabolites as a result of consuming PP-SDF (More in MSDF group) may regulate lipid metabolism.

In conclusion, our findings showed that PP-SDF intervention resulted in significant metabolic, transcriptional, and gut microbiota changes, which were favorable for the amelioration of obesity. PP-SDF intake reduced body weight and blood lipid levels and decreased inflammation levels in adipose and liver tissues in HFD-fed C57BL/6J mice. PP-SDF regulated lipid metabolism by enriching beneficial bacteria such as *Lachnospiraceae_*UCG-006, *Akkermansia, Coriobacteriaceae, Bifidobacterium*, and *Faecalibaculum*. Isobutyryl carnitine and dioscoretin were the key substances to regulate lipid metabolism and lipid metabolism was the main metabolic pathway. These results suggested that PP-SDF could be a beneficial supplement for the prevention of obesity, caused by high-fat diets and provided a basis for further studies on the specific pathways for lipid metabolism regulation.

## Data availability statement

The data presented in the study are deposited in the NCBI repository, accession number PRJNA881786.

## Ethics statement

The animal study was reviewed and approved by Laboratory Animal Ethics Committee of Chenguang Biotechnology Group Co. (ZY-LL-W21002).

## Author contributions

YJ and KM designed and performed the experiment and edited the manuscripts. ZW and JW helped with the animal experiment and metabolomics data analysis. CX helped with the animal experiment. JG and FS provided crucial help in 16S bacterial sequencing data analysis and language review. BC reviewed the manuscript and language correction. JG and YS helped design the experiments, reviewed the manuscript, as the guarantor of this work, had full access to all data in the study, takes responsibility for the integrity of the data, and the accuracy of data analysis. All authors contributed to the article and approved the submitted version.

## Funding

This work was supported by the Natural Science Foundation of Hebei Province (C2021204061).

## Conflict of interest

The authors declare that the research was conducted in the absence of any commercial or financial relationships that could be construed as a potential conflict of interest.

## Publisher's note

All claims expressed in this article are solely those of the authors and do not necessarily represent those of their affiliated organizations, or those of the publisher, the editors and the reviewers. Any product that may be evaluated in this article, or claim that may be made by its manufacturer, is not guaranteed or endorsed by the publisher.

## Appendix A

Grouping information of C57BL-6J mouse obesity model in Supplementary Figure 1. The Effect of PP-SDF administration on Alpha-diversity and Beta-diversity of gut microbiota at 0-week are shown in Supplementary Figure 2. The Effect of PP-SDF administration on the composition of the gut microbiota in phylum and genus levels at 0-week in Supplementary Figure 3. And the differential metabolites were analyzed by PCA analysis at 0-week (Supplementary Figure 4).
