# Supplementary material for: Pear pomace soluble dietary fiber ameliorates the negative effects of high-fat diet in mice by regulating the gut microbiota and associated metabolites
Source: Front Nutr. 2022 Oct 20;9:1025511. doi: 10.3389/fnut.2022.1025511 (PMC9633104; doi:10.3389/fnut.2022.1025511)
Supplement: Supplementary file 1 [file Table_1.DOCX]

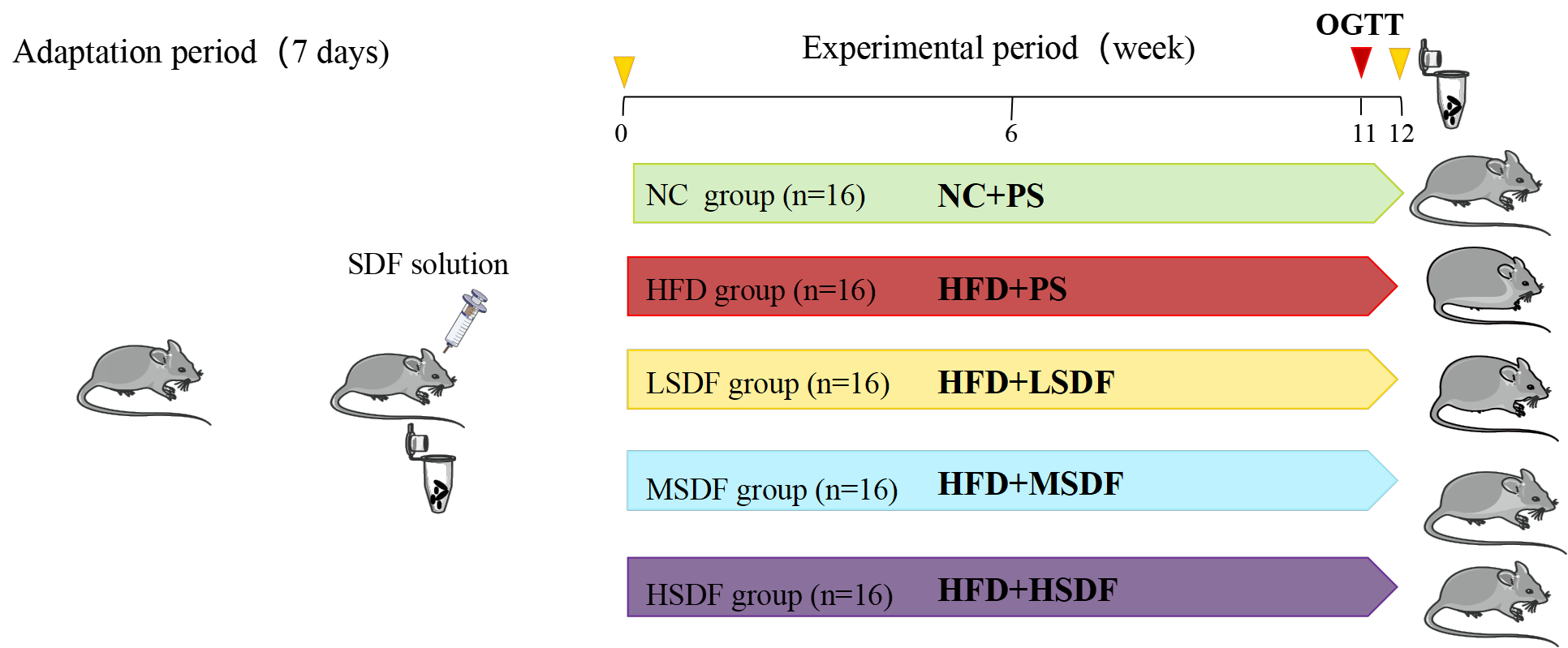


**S.1.Grouping information of C57BL-6J mouse obesity model.** The adaptation period took 7 days. Ninety C57BL-6J mice were randomly divided into 5 groups with 16 mice in each group. Normal control group (NC): Oral administration with an equivalent volume of 0.9% physiological saline (PS); high-fat-diet induced group (HFD): Oral administration with an equivalent volume of 0.9% physiological saline; low-dose soluble dietary fiber group (LSDF): oral administration with an equivalent volume of SDF (1g/kg·BW); medium-dose soluble dietary fiber group (MSDF): Oral administration with an equivalent volume of SDF (3g/kg·BW); high-dose soluble dietary fiber group (HSDF): oral administration with an equivalent volume of SDF (5g/kg·BW).Yellow triangle up solid: sampling sites for 16S rRNA sequencing; red triangle up solid: sampling sites for OGTT.


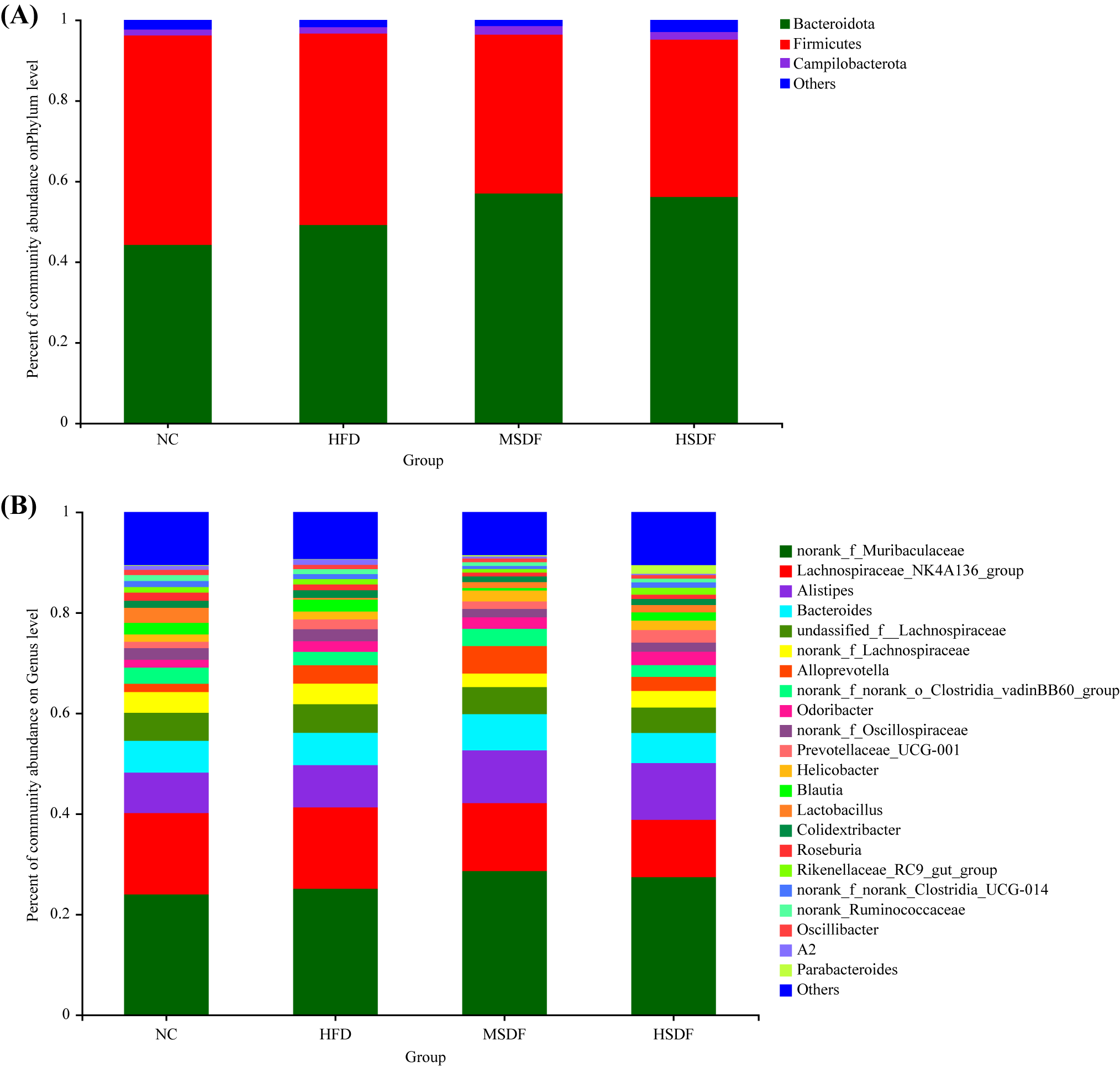


**S.2. Effect of PP-SDF administration on the composition of the gut microbiota in phylum and genus levels. (A)** At the 0-week phylum level. **(B)** At the 0-week genus level.


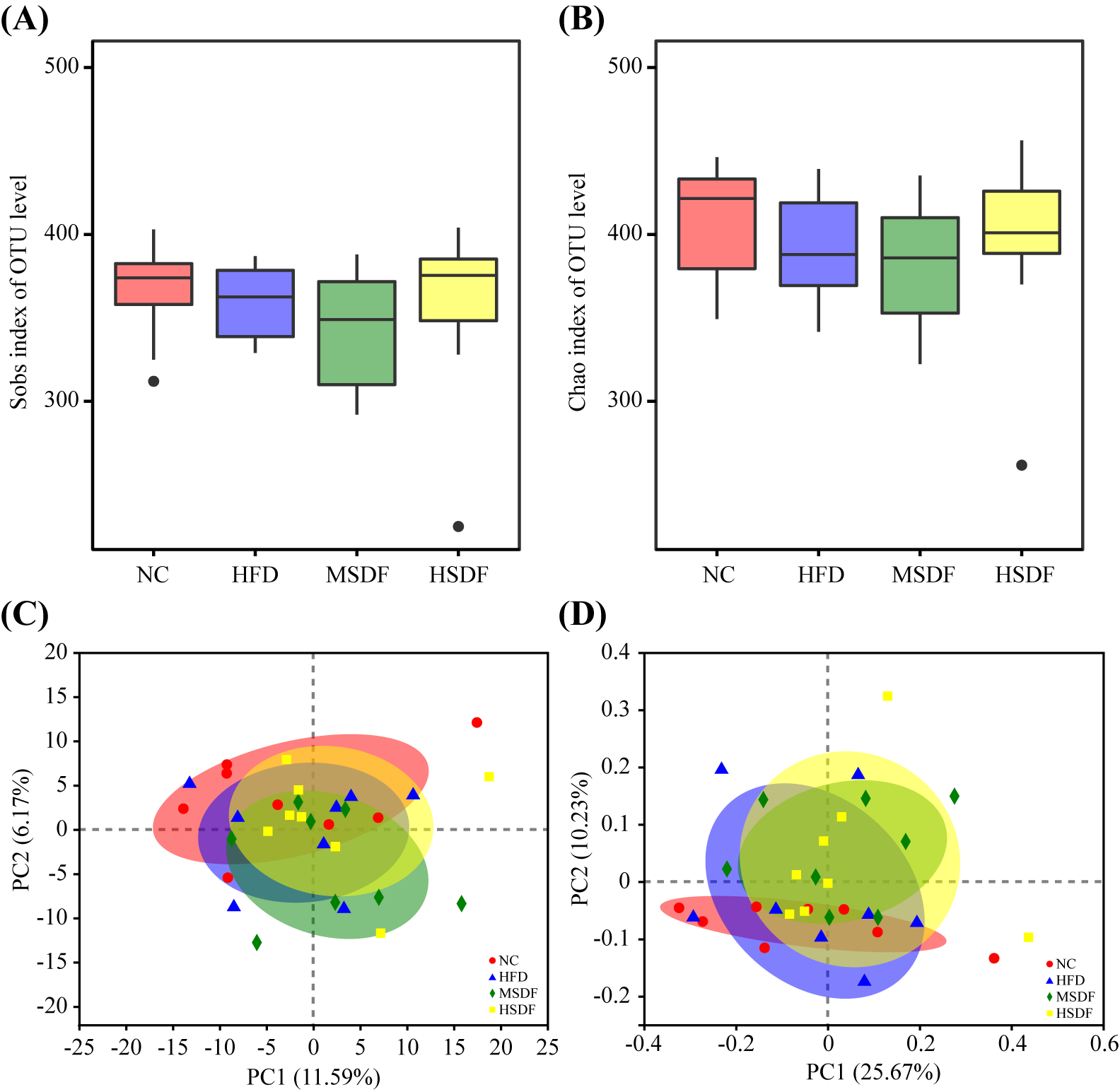


**S.3. Effect of PP-SDF administration on Alpha-diversity and Beta-diversity of gut microbiota. (A)** Sobs index at 0-week. **(B)** Chao index at 0-week. **(C)** PCA of bacterial community composition at 0-week. **(D)** PCoA plot of weighted UniFrac distance at 0-week.


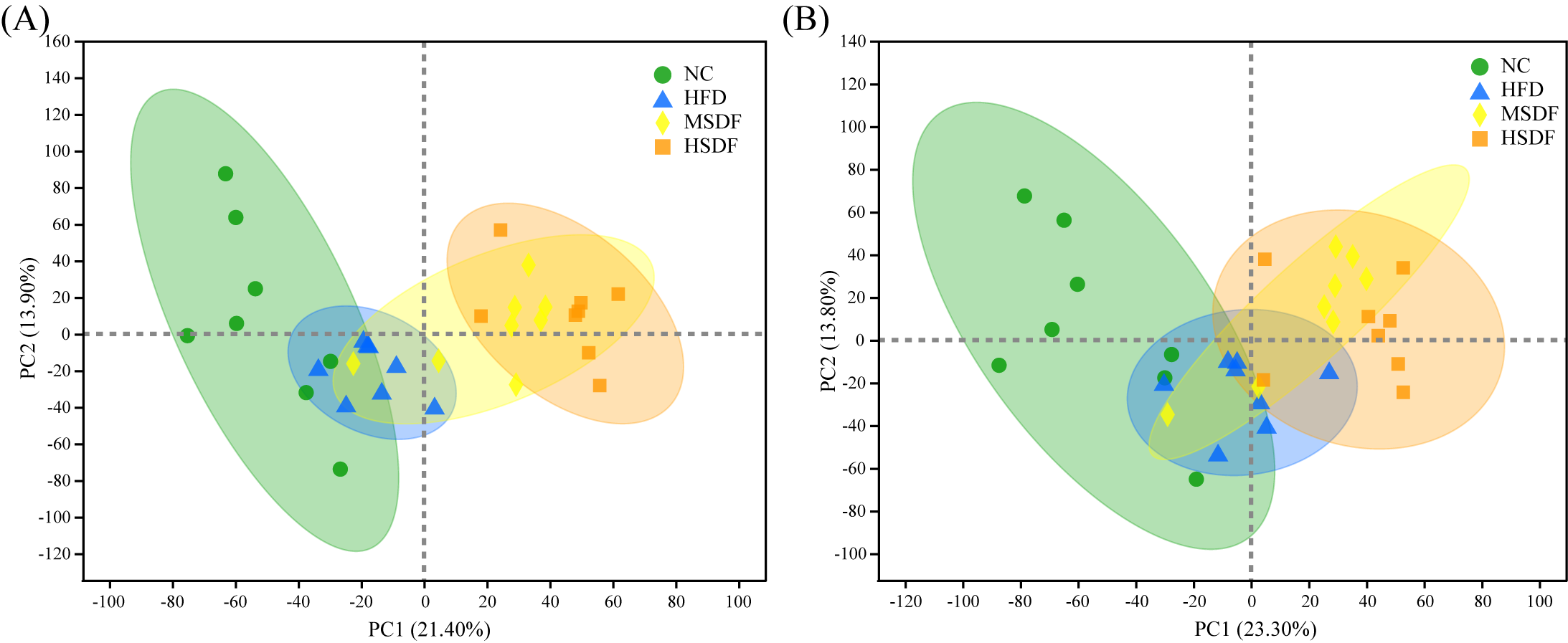


**S.4. PCA analysis between the four groups at the 0-week.** **(A)** PCA between four groups, positie ion mode. **(B)** PCA between four groups, negative ion mod
