# Supplementary figures and images for: Pear pomace soluble dietary fiber ameliorates the negative effects of high-fat diet in mice by regulating the gut microbiota and associated metabolites
Source: Front Nutr. 2022 Oct 20;9:1025511. doi: 10.3389/fnut.2022.1025511 (PMC9633104; doi:10.3389/fnut.2022.1025511)

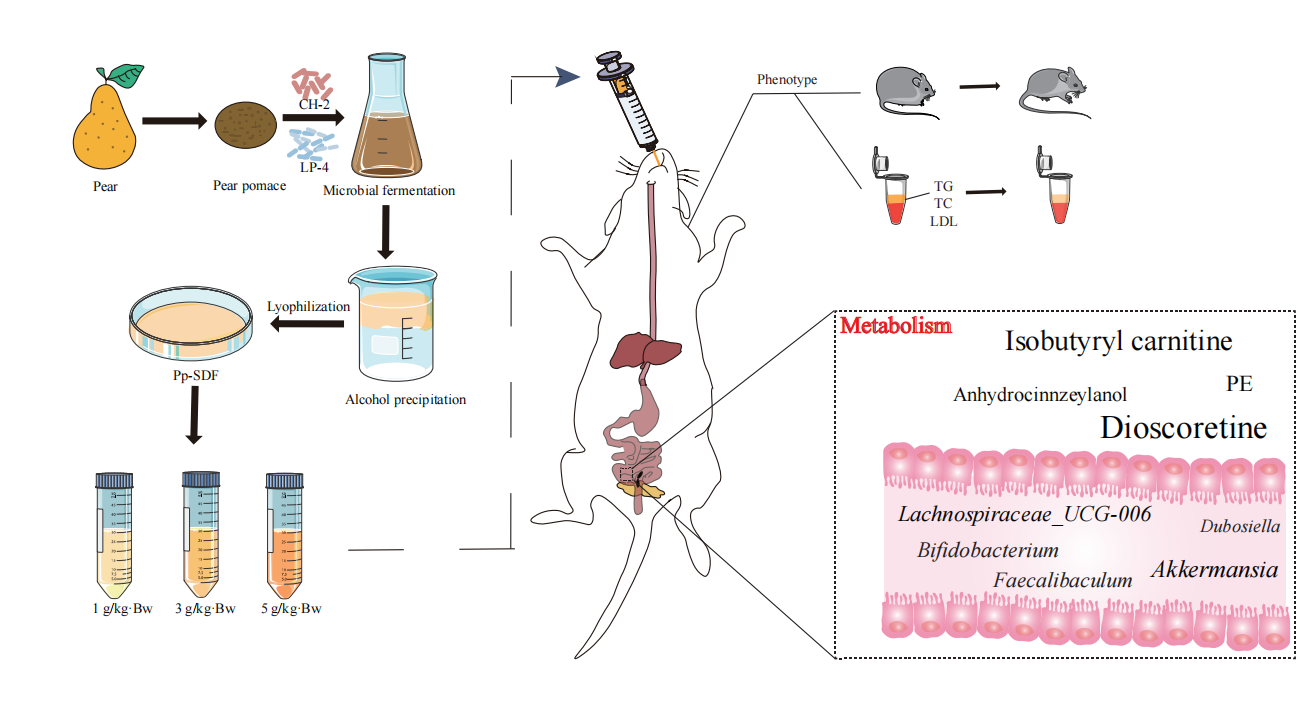

Supplement: Supplementary file 4 [file Image_1.TIF]
